# Novel *In Vitro* Study to Assess Microbial Barrier Properties of Polyurethane-Based Tissue Adhesives in Comparison to the Gold Standard Dermabond®

**DOI:** 10.1155/2022/5249214

**Published:** 2022-10-18

**Authors:** Yalda Mirzaei, Kerstin Hagemeister, René H. Tolba, Julia Steitz

**Affiliations:** ^1^Institute for Laboratory Animal Science, Faculty of Medicine, RWTH Aachen University, Aachen, Germany; ^2^Adhesys Medical GmbH, Aachen, Germany

## Abstract

Tissue adhesives as a physical barrier to microorganism penetration provide an alternative method with many advantages for wound closure in surgical settings compared to the clinical standard. This raises the need of developing and conducting *in vitro* methods that are sensitive and reproducible to assess their microbial barrier properties. In this study, three different polyurethane-based tissue adhesives with different physicochemical properties were evaluated in comparison to Dermabond® as a clinical gold standard for topical wound closure. Here, physicochemical properties varied in lactide concentration, viscosity, processing, and the full polymerization time. To evaluate the microbial barrier function, a 5 *μ*l aliquot of *E. coli Lux* inoculum containing at least 1 × 10^9^ CFU/ml was applied to the surface of each test adhesive and sterile filter paper as the control that was placed on an agar plate and incubated at 37°C. Plates were observed for bacterial growth (morphology), the adhesion of the adhesive/filter paper, and bioluminescence after 24, 48, and 72 hours. The data presented in this *in vitro* model indicated that polyurethane-based tissue adhesives with lactide concentration ≥ 5% provided a suitable barrier against microbial penetration with 95% confidence of 99% efficacy for 72 h along with Dermabond®. Interestingly, the here described method was able to discriminate between the different physicochemical properties showing a better microbial barrier function with increasing lactide concentration of the adhesive. Overall, the results of this study showed the noninferiority between Dermabond® and the two abovementioned polyurethane-based tissue adhesives.

## 1. Introduction

Worldwide public health initiatives have traditionally focused on the control of infectious disease, health promotion, and disease prevention including the proper treatment of wounds. Here, suturing, surgical tapes, staples, and ligating clips are currently the main clinical standards for wound closure as an essential and often expensive part of the health system [1]. However, tissue adhesives as a physical barrier to microorganism penetration provide a new option to ease the use of those invasive techniques and have gained increasing attention [2]. Tissue adhesives if used as topical wound closure devices benefit from less risk of needle stick injury and accordingly decrease the rate of suture-tract infections and fluid or gas leakage. They also offer a fast and painless alternative method for wound closure that is easier to apply and remove [3, 4]. Information on closure strength, microbial protection, and other physicochemical properties of tissue adhesives allows healthcare practitioners to decide which product will provide better clinical outcomes. Data revealed that surgical site infections (SSIs) are among the main causes of morbidity, prolonged hospitalization, and death in many surgical procedures [5–7]. Therefore, preventing wound infection and enhancing the wound healing process are of great interest. The wound healing process naturally occurs in four complex phases: hemostasis, inflammation, proliferation, and remodeling. This mechanism involves several orderly events happening simultaneously [8]. Here, new methods such as sinusoidal and pulsed electromagnetic fields to accelerate the wound healing process and tissue regeneration have been tested [9–12]. Other studies suggested the benefit of applying local antiseptics such as chlorhexidine (CHX) to reduce the postsurgical bacterial load and host inflammatory response [13–16]. CDC guidelines for the prevention of SSIs recommend that a wound should be covered with a sterile wound closure device for at least 24-48 hours to provide a barrier against microorganism entry into the surgical site and reduce the risk of infection [17]. Considering this, an acceptable microbial barrier property for a tissue adhesive as a wound closure device is a fundamental prerequisite. Despite having some limitations such as low bonding under wet conditions and not showing enough cytocompatibility, fibrin glue, gelatin-resorcin formaldehyde/glutaraldehyde glues, and cyanoacrylate glue could receive approval for clinical use [18–20]. Cyanoacrylate tissue adhesives first synthesized in 1949 are made from alkyl *α*-cyanoacrylates [21]. The difference between various cyanoacrylate tissue adhesives is based on the length of their alkoxy carbonyl (-COOR) chain [22]. Dermabond® as a well-known example of long-chain cyanoacrylates with an 8-carbon alkyl chain forms a strong, good-tolerated seal as a bridge over the wound edges that enhance patient comfort and cosmetic outcomes [21–23]. It is supported by a large and diverse body of published literature, including 53 randomized controlled trials (RCTs) [24–26]. In one *in vitro* study, Dermabond® adhesive was demonstrated as a microbial barrier with 99% protection for at least 72 hours against organisms that are commonly responsible for SSIs [27]. These data offer a strong rationale to choose Dermabond® as the clinical gold standard for our study. Polyurethane-based tissue adhesives as entirely synthetic surgical adhesives are made of a polyurethane prepolymer and an amine-based curing agent. As soon as the prepolymer mixes with the curing agent, the poly-addition reaction begins and will be completed after a few minutes. Then, the components adhere quickly to the tissue via mechanical and physical mechanisms. Having a honey-like viscosity, they can be used to hold approximated skin edges of wounds in less invasive surgical incisions [28]. The purpose of the work described herein was to evaluate three different polyurethane-based tissue adhesives with different physiochemical properties for their microbial barrier function compared to Dermabond® using an *in vitro* method established in our previous study [29].

## 2. Materials and Method

### 2.1. *E. coli Lux* Culture

*Escherichia coli Lux* (kindly provided by Dr. Timo Schwandt) was cultured in sterile standard I nutrient broth medium (NB) (Carl Roth GmbH + Co. KG, Karlsruhe, Germany) containing 100 *μ*g/ml ampicillin (ampicillin sodium salt, Carl Roth GmbH + Co. KG, Karlsruhe, Germany) and incubated at 37°C in an incubator shaker with 190 rpm (Stuart Reciprocating Shaker SSL2, Cole-Parmer GmbH, Wertheim, Germany) as described before [29].

### 2.2. Optical Density Determination and Titration Assay

Overnight *E. coli Lux* cultures were characterized first by the optical density measurement using a spectrophotometer at the wavelength of 600 nm (BioPhotometer®, Eppendorf, Hamburg, Germany). Then, the cultures with an OD_600_ value of around 3 were used to perform a titration assay with the serial dilution of 1 : 2 on a black 96-well plate, and bioluminescence signals were measured using the IVIS® Lumina XR II imaging system (Caliper Life Sciences, Inc., Hopkinton, MA, USA).

### 2.3. Colony-Forming Unit (CFU) Determination

To determine the colony-forming unit (CFU)/ml, 50 *μ*l of serial dilutions (10^4^, 10^5^, and 10^6^) of the overnight *E. coli Lux* culture was cultured on Tryptone Blood Sheep Soy Agar (TBSA) plates (Oxoid™ Deutschland GmbH, Wesel, Germany). Plates containing 30–300 colonies were counted to calculate the final bacterial concentration as CFU/ml values.

### 2.4. The Adhesive Film Preparation

Dermabond® tissue adhesive (Dermabond; Ethicon US LLC, Somerville, New Jersey; LOT MKJ833) was applied directly to the surface of agar plates by dispensing the liquid through the applicator tip in an area approximately in dimensions of 1 × 1 cm. Three different variations of the new polyurethane-based tissue adhesive named, respectively, AM1, AM2, and AM3 (Adhesys Medical GmbH, Aachen, Germany) were provided in a single-use two-chambered ready-to-use syringe, each with different physicochemical properties. Lactide as the cyclic di-ester of lactic acid defines the pH level of the, adhesives whereas raw materials based on a diamine (DCA) or triamine (TCA) work as the hardener. The prepolymer (Polyol) was three functional (Tri), and the ratio of DCA/TCA was 85/15 percent in all variations. Each tissue adhesive was precured as a layer on the inside of the sterile aluminum pouch. The film strips had approximately 2 mm thickness and were allowed to polymerize under a laminar flow hood for 3-4 minutes. The required time was decided based on the processing time (pot life) and tack free time (TFT) for each tissue adhesive. Then, they were cut with a sterile scalpel into 1 × 1 cm squares and carefully placed in the center of a TBSA agar plate. 1 × 1 cm squares of sterilized filter papers served as a control. A total number of 10 test articles for Dermabond®, AM1, AM2, AM3, and the control for each time point were employed.

### 2.5. Inoculation

The surface of each test article was inoculated with 5 *μ*l of a 100-fold dilution of *E. coli Lux* culture containing at least 1 × 10^9^ colony-forming units (CFU/ml). All test and control plates were incubated for 3 days at 37°C. Plates were observed for bacterial growth (morphology), the adhesion of the adhesive/filter paper, and measurement of bioluminescence using the IVIS imaging technique every 24 hours.

### 2.6. Imaging Protocol

Bioluminescence imaging was performed using an IVIS® Lumina XR II imaging system (Caliper Life Sciences, Inc., Hopkinton, MA, USA). For the bioluminescence measurements, plates were placed in the specimen chamber, and photon emission was measured with the following settings: binning: 8, aperture (f/stop): 1, field of view (FOV): D, the subject height: one centimeter, and the exposure time ranging from 0.5 seconds to 1 min depending on the bioluminescence intensity signal. All settings and controls that are required for image acquisition and processing were automated using Living Image® 4.7.3 software (RRID: SCR_014247).

### 2.7. Data Analysis and Statistical Methods

Data analysis was performed via the Living Image® 4.7.3 software, Microsoft Excel (RRID: SCR_016137) and Graph Pad Prism version 8.1.0 (San Diego, California, USA, RRID: SCR_002798). The total flux values of the background were subtracted from the values of test regions followed by the calculation of the mean value and standard deviation of each replicate. The data obtained from all measurements were then converted to CFU/ml values according to the correlation equation and were subsequently expressed as a mean ± standard deviation. Multiple *t*-tests were performed for each experimental round to determine the *p* value using the two-stage linear step-up procedure of Benjamini, Krieger, and Yekutieli, with *Q* = 1%. For each time point, the difference between test groups and control groups was analyzed individually (3 *t*-tests for each round of experiments) without assuming a consistent SD. The effects were considered to be significant if the *p* value was < 0.05. The percent maintenance of the microbial barrier against bacterial growth underneath the tissue adhesives compared to the control group was also determined using the following equation: [(Ac–At)/Ac] × 100 where Ac is the average of total flux values from 10 replicates of the controls and At is the average of total flux values from 10 replicates of the tissue adhesives. The mean percent maintenance of the 10 replicates was calculated afterward and considered as the total percent maintenance for each tissue adhesive.

## 3. Results

**Figure 1:**
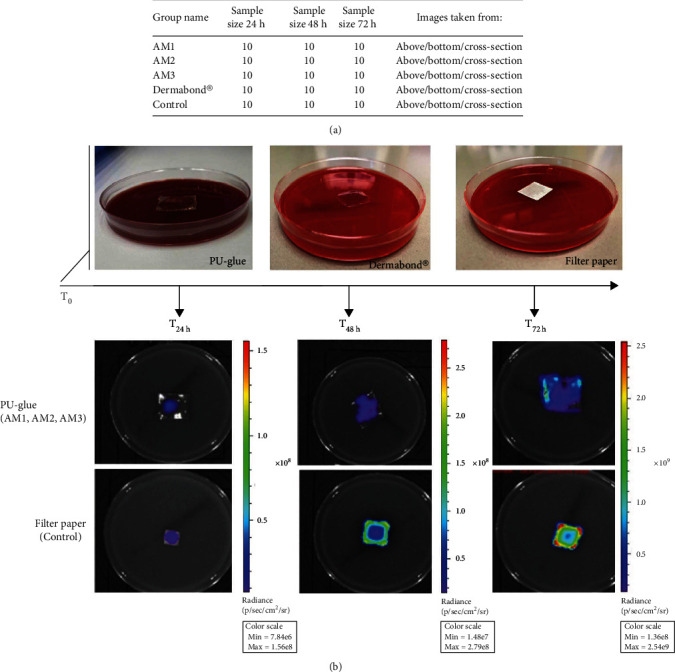
Experimental design. (a) Experimental design and group assignment. (b) *T*_0_: characterization, inoculation, and application of *E. coli Lux* on the test articles, representative images of polyurethane-based tissue adhesive (PU-glue: AM1, AM2, and AM3), Dermabond®, filter paper (control) on the agar plate. *T*_24h_, *T*_48h_, *T*_72h_: morphology observation and bioluminescence measurement using IVIS imaging system, representative images of PU-glue (upper row) and filter paper (lower row) from above at each time point.

A harmonized protocol for the experiment was planned to avoid intervention bias as shown in Figure 1. Of the 80 test articles evaluated for different adhesives, all of them retained their integrity as a microbial barrier for 72 h assessed by visual observation. In all control groups and test groups, bacteria growth was detected by observing bacteria colonies after 24 h, 48 h, and 72 h. For AM1, AM2, and AM3 groups as test groups, bacteria overgrowth around the adhesive area on plates was observed at 48 h and 72 h, whereas in the Dermabond® group, no bacteria overgrowth around the Dermabond® edges was noted at different time points. After morphology observation, for each test and control plate at each time interval, three different images were obtained: (i) from above, which measured the whole surface of adhesives/filter papers; (ii) from the bottom site after removal of the adhesives/sterile filter papers, which measured only the area below; and (iii) cross-section which measured the half-cut surface of adhesives/filter papers placed perpendicularly on the TBSA plate. The bioluminescence measurement values in photon per second obtained from titration assay before inoculation of *E. coli Lux* were correlated with the colony-forming units' values. The results in all four rounds of the experiment clearly showed that bacteria cultures with an optical density (OD_600nm_) around 3, based on our previously published method, will result in comparably similar CFU/ml values with a positive correlation with the bioluminescence signal as photon per second. In all the experiments, the *p* value and the degree of freedom were calculated to determine if the slope is significantly nonzero. The *p* values in all experiment rounds were less than 0.0001; thus, the assumptions of a linear relationship between bacteria viability and luminescence measurements have been met. The *R* square values were >0.92 for all rounds of the experiment indicating a high correlation between the measured bioluminescence and the CFU/ml counts (data are not shown). In the presented study, three different polyurethane adhesives differing in lactide, polyol concentration, the raw material used (DCA/TCA), the processing time (pot life), and polymerization time (TFT) were tested. As shown in Table 1, for AM1, the initial viscosity was less than AM2 and AM3; therefore, it had a more liquid form. Accordingly, the pot life, the length of time in which multiple part coatings can be applied before the mix is unsuitable for application, was higher in AM1 compared to AM2 and AM3 (75, 60, and 60 seconds, respectively). However, AM2 had a less TFT, the time at which the polymerization is fully done and the adhesive has sufficient protection without being disrupted or damaged due to contact or handling, in comparison to AM1 and AM3.

**Figure 2:**
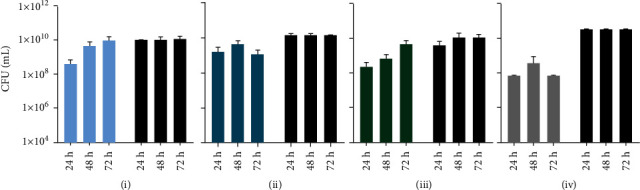
Results of bioluminescence measurement taken from above converted into CFU/ml after inoculation with 5 *μ*l of a 1 : 100 dilution of *E. coli Lux* culture at indicated time points: (a) AM1, (b) AM2, (c) AM3, and (b) Dermabond®. Values are means, and error bars represent SD.

**Figure 3:**
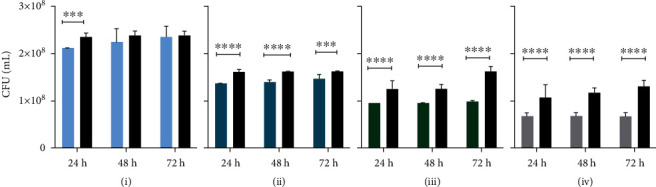
Results of bioluminescence measurement taken from bottom site converted into CFU/ml after inoculation with 5 *μ*l of a 1 : 100 dilution of *E. coli Lux* culture at indicated time points: (a) AM1, (b) AM2, (c) AM3, and (d) Dermabond®. Values are means, and error bars represent SD. ^∗∗∗^ for *p* ≤ 0.001 and ^∗∗∗∗^ for *p* ≤ 0.0001.

**Figure 4:**
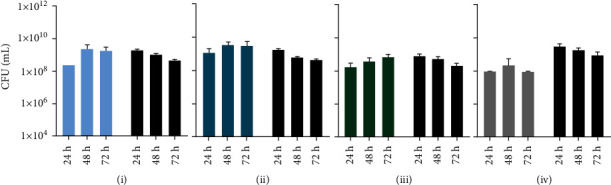
Results of bioluminescence measurement taken from cross-section converted into CFU/ml after inoculation with 5 *μ*l of a 1 : 100 dilution of *E. coli Lux* culture at indicated time points: (a) AM1, (b) AM2, (c) AM3, and (d) Dermabond®. Values are means, and error bars represent SD.

**Figure 5:**
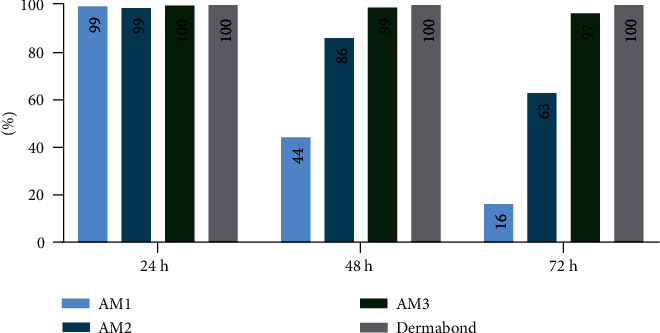
Graphical illustration (bar chart) of the percent maintenance of the microbial barrier against bacterial growth for AM1, AM2, AM3, and Dermabond® based on total flux values upon different time points after removal of the tissue adhesives.

In general, bacterial growth measured by bioluminescence imaging was significantly reduced at 24 h, 48 h, and 72 h time points when measured from above indicating a slow bacterial growth (Figure 2). At the bottom site, the results showed that at 24 h in all four test groups (AM1, AM2, AM3, and Dermabond®), the *p* value between test and control groups after removal of the adhesives and sterile filter papers was less than 0.05 (*p* < 0.000001). Thus, all tissue adhesives provided a suitable barrier against microbial penetration with 95% confidence for 24 h in this *in vitro* model. The same calculations at 48 h and 72 h demonstrated that the *p* values between AM1 and the control group were higher than 0.05 at 48 h and 72 h. Thus, the data presented in this set showed that AM1 tissue adhesive did not provide an effective barrier for microbial penetration for 48 h and 72 h. At 48 h, the differences between test and control groups were significant in AM2, AM3, and Dermabond® groups. These findings highlighted that AM2, AM3, and Dermabond® were effective barriers against bacteria penetration for 48 h in this setup. At 72 h, AM2 and AM3 polyurethane-based tissue adhesives showed *p* values of less than 0.05, representing a significant difference between test and control groups along with Dermabond® (Figure 3). Analysis of the cross-sections showed significant differences between all four test groups and control groups at 24 h, 48 h, and 72 h similar to the measurement from above counting all bacteria on the test material (Figure 4). The percent maintenance values of the microbial barrier against bacterial growth underneath the AM1 adhesive group compared to the control group were 99.41%, 44.28%, and 16.09% at 24 h, 48 h, and 72 h, respectively. The same calculation for AM2, AM3, and Dermabond® test groups showed values of 98.72%, 86.11%, and 62.88% for AM2; 99.77%, 98.87%, and 96.52% for AM3; and 99.99%, 99.98%, and 99.99% for Dermabond® at 24 h, 48 h, and 72 h, respectively (Figure 5).

## 4. Discussion

Traditional methods in surgical procedures for wound closure or internal implant fixation can cause a significant mismatch between the tissue and the fixation, leakage, infection, and additional trauma due to the puncturing [30, 31]. Tissue adhesives can potentially overcome these limitations and provide additional benefits [32, 33]. However, evaluation of microbial barrier properties of tissue adhesives is one of the important steps before using them in preclinical and clinical practices regarding the risk of microbial contamination and surgical site infection. Before a barrier to outside elements is formed by basal cells within 48 h, the wounded tissue is solely dependent on the wound closure device to maintain its integrity due to the lack of appropriate tensile strength [34]. Hence, any wound closure device such as tissue adhesives that act as an effective barrier against outside elements for up to 72 h can provide sufficient time for the natural wound healing mechanism to proceed. The conventional *in vitro* test for evaluating the microbial properties of tissue adhesive is based on the production of organic acids by actively growing bacteria that results in a changing colour in the agar plate as an indication of a breach on the adhesive layer [27]. The herein-described *in vitro* test method used the bioluminescence imaging technique to detect and measure a certain wavelength of light produced by metabolically active growing bacteria as described before [29]. By employing an appropriate control group, a true penetration could be distinguished from random contamination, and a rational comparison would be obtained. In this study, we have used Dermabond® tissue adhesive as a reference for topical wound closure, and our results supported the hypothesis of the previous studies that it provides microbial barrier protection for at least 72 hours [27]. The data also proved that AM2 and AM3 tissue adhesives are an effective barrier to microbial penetration with 95% confidence for 72 h. One study showed that Dermabond® has a bactericidal property against Gram-positive bacteria [35]. It is mentioned that this mechanism of action is due to the strong electronegative charge on the cyanoacrylate monomer that reacts with the positively charged carbohydrate capsule of Gram-positive organisms [36, 37]. The polyurethane-based tissue adhesive was previously evaluated in an *ex vivo* study showing good tensile strength in suture-less microsurgical anastomoses [38]. Other *in vivo* studies also showed that it can be a good candidate as a reinforcement for blood vessel anastomoses in a secure and less traumatic manner [28, 39]. For the polyurethane-based tissue adhesives tested herein, all three variations have the same prepolymer base but with different lactide percentages that can play an important role in changing the pH level of the adhesives (2%, 5%, and 8% for AM1, AM2, and AM3, respectively). As it is known, the acidic pH level can have an effect on killing or at least slowing down the growth of bacteria. Hence, one reason that AM3 and AM2 worked better than AM1 could be due to their higher lactide percentage. Moreover, AM3 also showed better microbial barrier properties compared to AM2 which has less lactide percentage (8% compared to 5%, respectively). While the polyurethane-based tissue adhesives showed promising results in providing a microbial barrier and suture reinforcement [28, 36, 38, 39], further optimization can be achieved by assessing new formulations to find the best formulation, time, and frequency of use as well as to elucidate the possible side effects. The main goal of research on new noninvasive methods in wound treatment is to evaluate them as a suitable means to help the natural wound healing process, reducing stress, pain, discomfort, and infection rate. In this framework, some studies proclaimed the benefit of using topical antimicrobial agents to inhibit bacteria plague formation [14–16]. Disinfection with chlorhexidine digluconate gel in a human study showed a reduction in the host inflammatory response and consequently marginal bone loss [13]. However, the short-term application limited the use of such agents [16]. Some other studies discussed the negative and positive impacts of exposure to electromagnetic fields (EMFs) on wound repair processes [40, 41]. The effect of low-frequency sinusoidal electromagnetic field (SEMF) and low-frequency pulsed electromagnetic field (PEMF) resulted in favor of wound healing on an oral model [9]. While many papers supported the benefit of using these treatments [9–12], their potential negative effects should be also considered [42–44]. Nevertheless, it can widen the scope of evaluating the combined use of noninvasive treatments such as tissue adhesives and topical disinfectants to reduce wound healing time and SSIs as the next step.

## 5. Limitations

The here presented novel *in vitro* method has some limitations that need to be addressed. The method requires specific equipment, and it is important to use the same settings (exposure time, the distance from lens to the sample, and subject height) and bacterial stocks to generate reproducible results among research labs. Moreover, the resolution and sensitivity of the measurement are not high enough to discriminate between the bacterial growth on the material itself and the bacterial growth through it. Therefore, the data from cross-section images are not suitable for assessing the microbial barrier function, and only the bacterial growth below the tissue adhesive should be considered a positive penetration event. Another limitation of our *in vitro* study is the lack of the holistic nature of wound healing (systemic and mechanical factors) by not simulating its major phases [40, 41]. Moreover, the method does not reflect the influence of the immune system, cell debris, and the complex interactions among different cell types (mesenchymal, hemopoietic, and epithelial) through the wound healing process [45–47]. Hence, to fully assess tissue adhesives, other wound repair models such as wound healing *in vitro* assays [46, 48], human three-dimensional skin models [49], and *ex vivo* [50–52] and in silico methods [47] that can mimic a microenvironment comparable to humans are recommended. In addition, the method does not allow for studying scar formation, skin aging, and the effect of wound movement. However, due to the absence of an inherent immune system, our *in vitro* assessment might be more sensitive compared to *in vivo* models meaning that higher numbers of bacteria would be required to generate an infection in an *in vivo* model [27, 29]. Therefore, *in vivo* models that provide direct analysis of a stimulus in the living human should be also considered.

## 6. Conclusion

The here presented *in vitro* model findings proved that AM2 and AM3 tissue adhesives built an effective barrier against microbial penetration with 95% confidence for 72 h and showed the noninferiority between Dermabond® and the two polyurethane-based tissue adhesives. In addition, data from AM3 tissue adhesive strongly demonstrated a similar microbial barrier strength as Dermabond® tissue adhesive at all-time points. Interestingly, the here described method was able to discriminate between the different physicochemical properties showing a better microbial barrier function with increasing lactide concentration of the adhesive. Mechanistically, the barrier property described above is also effective against other microorganism species responsible for surgical site infections. The here described novel *in vitro* method can be effectively used to evaluate microbial properties of different tissue adhesives alone or in combination with other methods. Considering that our *in vitro* method cannot investigate cell interaction or the role of the immune system in the wound healing process, additional *in vitro*, *ex vivo*, or *in vivo* studies might be used to fully test the biocompatibility of new adhesives.

## Figures and Tables

**Table 1:** Physicochemical properties of three different polyurethane-based tissue adhesives. Polyol is the prepolymer and Tri stands for three functional, DCA is the raw material based on a diamine, and TCA is the raw material based on a triamine. Pot life is processing time, and TFT is fully polymerization time for each adhesive.

Tissue adhesive	Lactide	Polyol	DCA/TCA	Pot life (second)	TFT (second)	Initial viscosity (mPa-s)
AM1	2%	204, tri	85/15	~75	~205	~4500
AM2	5%	206, tri	85/15	~60	~140	~7000
AM3	8%	208, tri	85/15	~60	~240	~5000

## Data Availability

The datasets analyzed during the current study are available from the corresponding author upon reasonable request.
